# Vitamin D-dependent Rickets Type 1A: Recognizing Clinical Clues to Avoid Delayed Diagnosis

**DOI:** 10.7759/cureus.116126

**Published:** 2026-09-12

**Authors:** Pedro Arthur da Rocha Ribas, Victoria Zeghbi Cochenski Borba

**Affiliations:** 1 Department of Internal Medicine, Serviço de Endocrinologia e Metabologia (SEMPR), Hospital de Clínicas da Universidade Federal do Paraná, Curitiba, BRA

**Keywords:** calcitriol, cyp27b1, delayed diagnosis, hereditary rickets, vitamin d-dependent rickets type 1a

## Abstract

Vitamin D-dependent rickets type 1A (VDDR1A) is a rare, inherited form of rickets caused by biallelic mutations in the CYP27B1 gene, leading to 1α-hydroxylase deficiency and impaired 1,25-dihydroxyvitamin D (1,25(OH)₂D) production. We report the case of a 25-year-old woman who was initially diagnosed at 12 months of age with "vitamin D-resistant rickets" and presented with motor delay, skeletal deformities, and a history of multiple orthopedic surgeries. At 25 years of age, etiologic evaluation identified a homozygous CYP27B1 p.Thr325Met variant, classified as likely pathogenic by the testing laboratory but previously reported as a variant of uncertain significance. The condition's rarity and its clinical overlap with more common forms of rickets hindered early diagnosis. Treatment with calcitriol and mineral supplementation was followed by sustained biochemical improvement over approximately one year, although established skeletal deformities persisted. This report underscores the importance of clinical and laboratory recognition, genetic testing, individualized management, and cautious interpretation of rare genetic variants.

## Introduction

Rickets is a metabolic bone disorder characterized by defective mineralization in individuals with open growth plates, resulting from disturbances in calcium and phosphate metabolism. Clinical manifestations include skeletal deformities, growth impairment, bone pain, and muscle weakness, with biochemical findings commonly including hypocalcemia, hypophosphatemia, elevated alkaline phosphatase (ALP), and secondary hyperparathyroidism [1].

Although nutritional rickets remains the most prevalent form worldwide, hereditary causes should be considered in patients with atypical clinical or biochemical features. Vitamin D-dependent rickets (VDDR) comprises genetic defects in vitamin D activation, action, or degradation. Vitamin D-dependent rickets type 1B (VDDR1B) results from CYP2R1 variants that impair hepatic 25-hydroxylation, while vitamin D-dependent rickets type 1A (VDDR1A) is caused by biallelic CYP27B1 variants that reduce renal 1α-hydroxylase activity and the conversion of 25-hydroxyvitamin D (25(OH)D) to 1,25-dihydroxyvitamin D (1,25(OH)₂D), or calcitriol. Vitamin D-dependent rickets type 2A (VDDR2A) results from vitamin D receptor (VDR) variants, vitamin D-dependent rickets type 2B (VDDR2B) involves impaired VDR-deoxyribonucleic acid (DNA) signaling, and vitamin D-dependent rickets type 3 (VDDR3) is caused by gain-of-function CYP3A4 variants that accelerate vitamin D metabolite inactivation [2,3]. In VDDR1A, reduced calcitriol synthesis impairs calcium and phosphate homeostasis despite normal or non-deficient 25(OH)D levels [2-4]. Affected children are generally normal at birth and typically present between six and 24 months of age with hypotonia, delayed motor development, growth impairment, and skeletal signs of rickets [4,5].

Because of its rarity and overlap with more common forms of rickets, VDDR1A may remain unrecognized, leading to progressive skeletal deformities and other preventable complications. We report a patient with longstanding rickets initially classified as "vitamin D-resistant rickets," in whom VDDR1A was diagnosed only in adulthood. This case highlights clinical and biochemical clues that may facilitate earlier recognition and appropriate treatment.

## Case presentation

A 25-year-old woman with short stature (1.49 m) was referred to our tertiary endocrinology center with a longstanding diagnosis of "vitamin D-resistant rickets," established at 12 months of age at another institution. For the following 24 years, she reportedly received vitamin D₃ and calcium supplementation. However, the exact formulations, doses, dose adjustments, adherence history, and serial biochemical results from this period were not available. There was no documented use of active vitamin D, including calcitriol, before referral to our center. The available records also did not indicate how treatment response had been monitored or why supplementation was continued without etiologic reassessment or therapeutic modification. Despite this prolonged treatment, she developed progressive skeletal deformities. Her parents and other known relatives were reportedly unaffected, with no family history of rickets or other skeletal disorders. There was no reported parental consanguinity or other known relatedness. Genetic testing was limited to the proband, and parental testing and segregation analysis were not performed. The major clinical events, diagnostic evaluation, treatment, and follow-up are summarized in Table 1.

**Table 1:** Clinical timeline of the patient's presentation, evaluation, treatment, and follow-up. ALP: alkaline phosphatase; PTH: parathyroid hormone; 25(OH)D: 25-hydroxyvitamin D; TRP: tubular reabsorption of phosphate; TmP/GFR: tubular maximum phosphate reabsorption to glomerular filtration rate; FGF23: fibroblast growth factor 23; 1,25(OH)₂D: 1,25-dihydroxyvitamin D

Age or period	Clinical events and management
12 months	Diagnosis of "vitamin D-resistant rickets" at another institution after early motor delay, hypotonia, wrist widening, and genu varum
Childhood and adolescence	Reported vitamin D₃ and calcium supplementation. Exact doses, adherence, monitoring, and serial laboratory results were unavailable. Progressive deformities developed, and eight orthopedic procedures were performed
12 years	Most recent orthopedic procedure: medial distal femoral epiphysiodesis of the right knee
25 years: referral	Referral to the tertiary endocrinology center with hypocalcemia, hypophosphatemia, elevated ALP and PTH, normal 25(OH)D, and severe longstanding skeletal deformities. Baseline TRP, ratio of TmP/GFR, FGF23, and 1,25(OH)₂D were unavailable
25 years: etiologic evaluation	Customized hereditary rickets panel identified a homozygous CYP27B1 c.974C>T (p.Thr325Met) variant, classified as likely pathogenic by the testing laboratory. Parental testing and segregation analysis were not performed
After etiologic evaluation	Conventional vitamin D₃ was discontinued. Calcitriol 0.25 mcg/day, elemental phosphorus 500 mg/day, and calcium carbonate 1,500 mg/day, equivalent to approximately 600 mg/day of elemental calcium, were prescribed
Approximately one year	Improvement in serum calcium, phosphate, ALP, and PTH, although PTH remained elevated. TRP was 88%. Clinical stability, preserved renal function, normal 24-hour urinary calcium, and no nephrolithiasis or nephrocalcinosis on ultrasonography were documented

During early childhood, she developed delayed motor milestones, hypotonia, marked genu varum, wrist widening, and intermittent bone pain. Despite continuous medical follow-up, the etiologic diagnosis remained unrecognized throughout childhood and adolescence, with worsening skeletal deformities requiring multiple orthopedic interventions.

**Figure 1:**
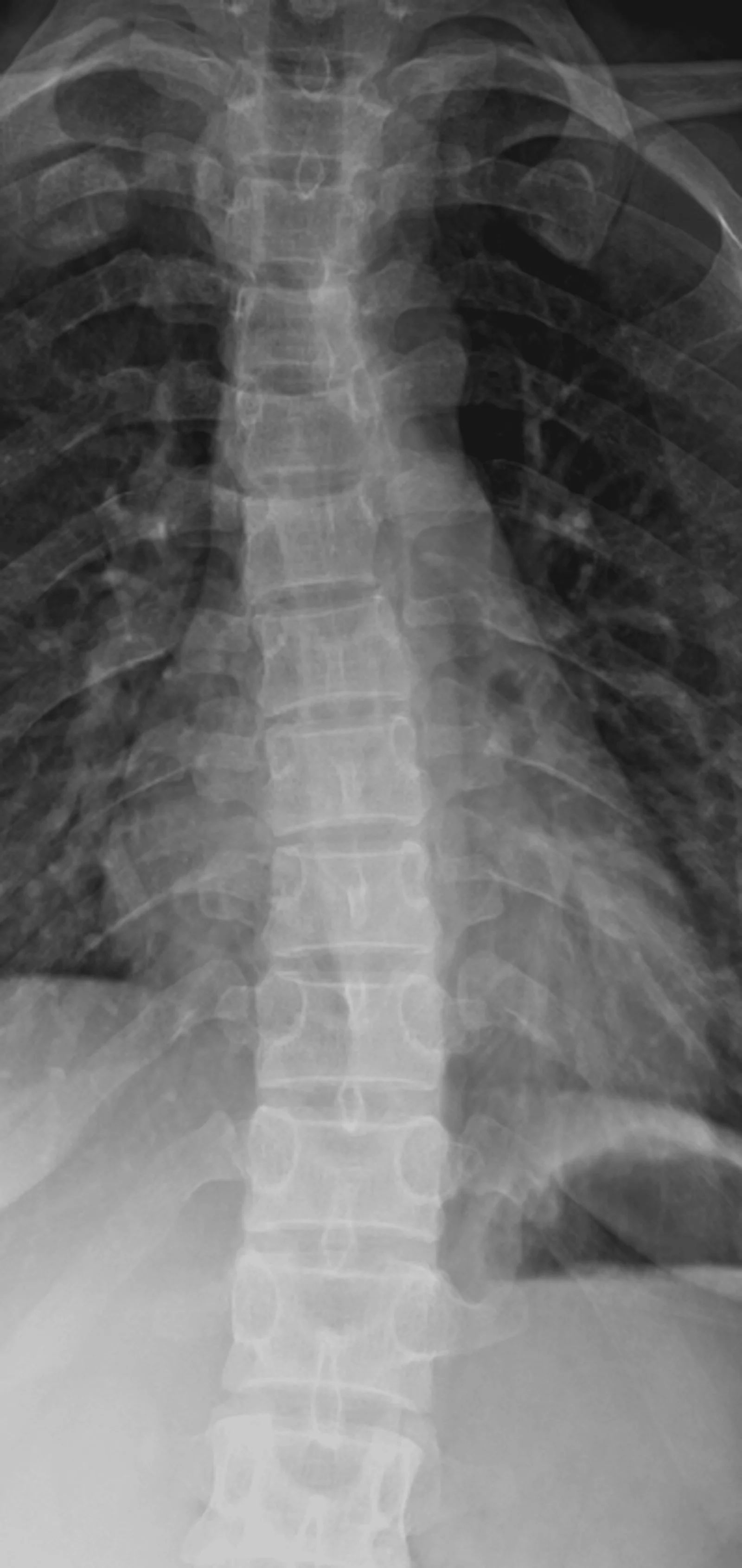
Thoracic radiograph demonstrating scoliosis.

**Figure 2:**
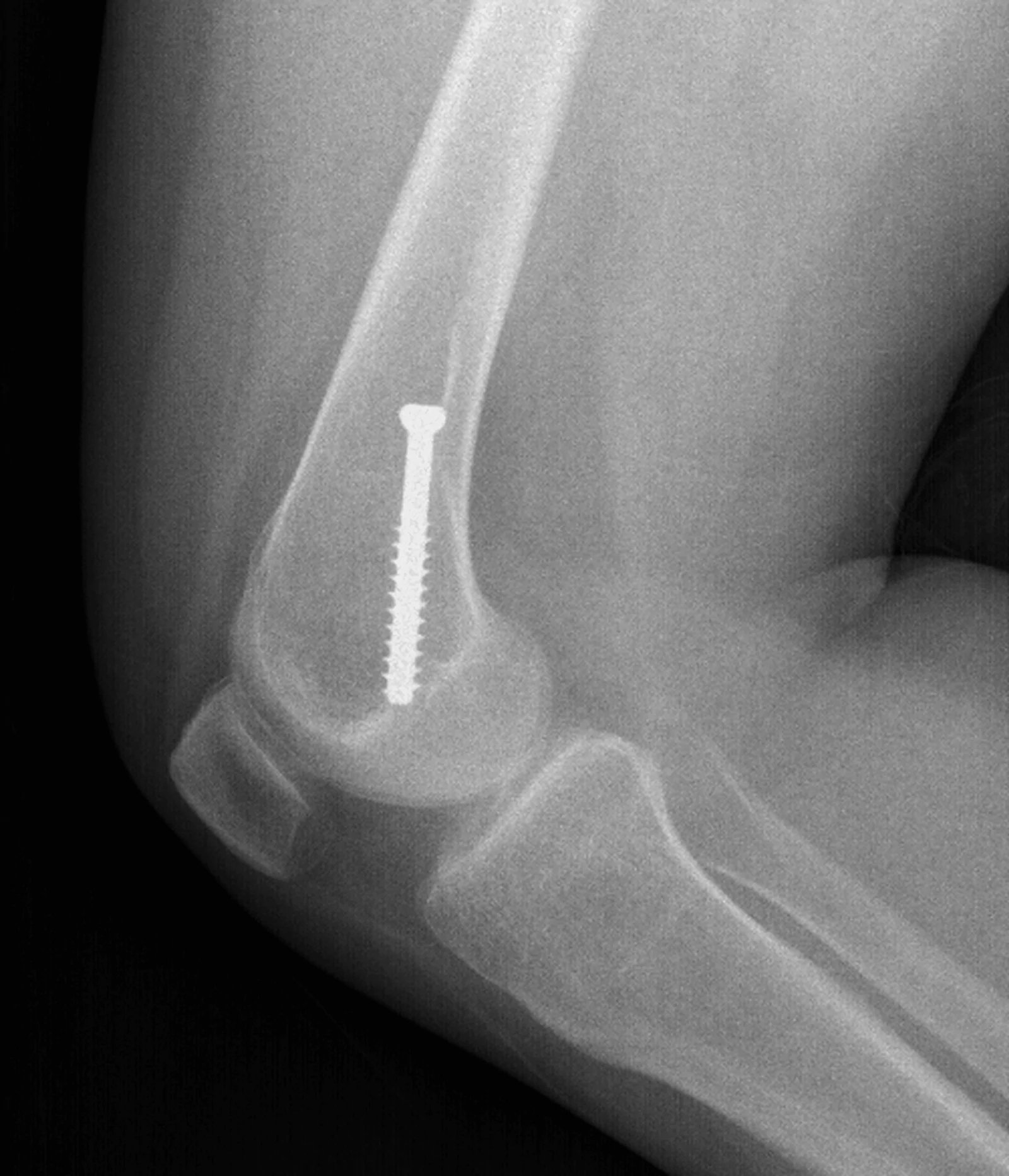
Lateral radiograph of the right knee showing medial distal femoral epiphysiodesis performed to correct angular deformity secondary to longstanding rickets.

At presentation, physical examination revealed short stature and severe longstanding skeletal deformities, including marked genu varum, wrist widening, and thoracic scoliosis (Figure 1), consistent with chronic metabolic bone disease. Owing to progressive lower-limb deformities, she underwent eight orthopedic procedures during childhood and adolescence. The most recent orthopedic procedure, performed at 12 years of age, was a medial distal femoral epiphysiodesis of the right knee to improve lower-limb alignment (Figure 2).

The laboratory results available at referral had been obtained during the patient's previous follow-up at the referring institution. They showed elevated ALP and parathyroid hormone (PTH), low serum calcium and phosphate, low 24-hour urinary calcium and phosphate excretion, and normal serum 25(OH)D levels (Table 2). Paired serum and urinary phosphate and creatinine measurements required to calculate tubular reabsorption of phosphate (TRP) and the ratio of tubular maximum phosphate reabsorption to glomerular filtration rate (TmP/GFR) were not available. Therefore, baseline renal phosphate handling could not be directly assessed. The low absolute 24-hour urinary phosphate value was not interpreted as excluding renal phosphate wasting because urinary phosphate excretion alone is influenced by serum phosphate and intake and does not quantify fractional tubular handling. Serum fibroblast growth factor 23 (FGF23) was not measured because this assay had not been included in the external evaluation and was not routinely available at our center. Although the hereditary rickets panel included the FGF23 gene, genetic analysis does not substitute for the measurement of circulating FGF23. The available biochemical profile was not typical of vitamin D deficiency rickets and raised suspicion of a genetic etiology. The combination of marked secondary hyperparathyroidism, hypocalcemia, and normal 25(OH)D levels was considered more compatible with impaired vitamin D activation than with a primary phosphate-wasting disorder.

**Table 2:** Initial laboratory findings at presentation to our service. ALP: alkaline phosphatase; PTH: parathyroid hormone; 25(OH)D: 25-hydroxyvitamin D

Test	Result	Normal reference range
ALP	286 U/L	35-105 U/L
Calcium	8.3 mg/dL	8.6-10.4 mg/dL
Phosphate	1.8 mg/dL	2.5-4.5 mg/dL
PTH	863 pg/mL	15-65 pg/mL
25(OH)D	27 ng/mL	20-50 ng/mL
Urinary phosphate	69.7 mg/24 hours	400-1,300 mg/24 hours
Urinary calcium	6.5 mg/24 hours	40-240 mg/24 hours

Because of the persistent clinical and biochemical abnormalities, etiologic investigation was initiated after referral to our tertiary endocrinology center. Measurement of 1,25(OH)₂D was not performed because of limited availability and high cost. Genomic DNA extracted from a buccal swab was analyzed using a customized next-generation sequencing (NGS) panel that included ALPL, CLCN5, CTNS, CYP27B1, CYP2R1, DMP1, ENPP1, FAH, FAM20C, FGF23, FGFR1, GNAS, KL, OCRL, PHEX, SLC34A1, SLC34A3, and VDR. Libraries were prepared using Illumina DNA Prep with Enrichment (Illumina, Inc., San Diego, CA, USA) and a customized version 4 target-capture panel (Twist Bioscience Corporation, South San Francisco, CA, USA) targeting coding exons and adjacent regions, followed by sequencing on an Illumina NovaSeq X Plus platform (Illumina, Inc., San Diego, CA, USA). Reads were aligned to the Genome Reference Consortium Human Build 38 (GRCh38) reference genome using Burrows-Wheeler Aligner Maximal Exact Matches (BWA-MEM). Small variants and insertions or deletions were identified using DeepVariant (Google Research, Google LLC, Mountain View, CA, USA), while copy number variants were assessed using ExomeDepth. Overall, 98.8% of targeted bases had a minimum coverage of 20 reads, with a mean depth of 72 reads. The analysis identified a homozygous CYP27B1 variant (GRCh38 chr12:57,764,540 G>A, c.974C>T, p.Thr325Met, ENST00000228606). The testing laboratory classified this variant as likely pathogenic according to its application of the American College of Medical Genetics and Genomics and Association for Molecular Pathology (ACMG/AMP) recommendations [6]. The laboratory reported that the variant had been observed in heterozygosity in two of approximately 807,000 individuals in population databases. Its assessment also considered conservation of Thr325, deleterious in silico predictions, previous identification in the homozygous state in a patient with rickets, three additional affected individuals identified in the laboratory's internal database, and concordance with the patient's phenotype. However, the report did not provide the individual ACMG/AMP criteria or a public database accession. No parental segregation or variant-specific functional analysis was performed. No additional pathogenic variants were identified in the other genes included in the panel.

Following clinical and molecular evaluation supporting VDDR1A, the previous vitamin D₃ regimen was discontinued, and treatment was optimized with oral calcitriol 0.25 mcg once daily, elemental phosphorus 250 mg twice daily (500 mg/day), and calcium carbonate 500 mg three times daily (1,500 mg/day of calcium carbonate, equivalent to approximately 600 mg/day of elemental calcium). Phosphorus was added because of the marked hypophosphatemia at referral. No dose changes were documented during the approximately one-year follow-up. The patient was monitored with clinical and biochemical assessments to evaluate response and detect potential complications, particularly hypercalciuria, nephrolithiasis, and nephrocalcinosis.

The patient has been followed at our center for approximately one year while receiving calcitriol and mineral supplementation. The available paired assessments at referral and at approximately one year showed that serum calcium and phosphate increased and ALP and PTH decreased (Table 3). Although improved, PTH remained markedly elevated, indicating incomplete biochemical control and the need for continued reassessment of calcitriol and calcium dosing. Twenty-four-hour urinary calcium increased during treatment, consistent with increased calcium availability following the correction of the calcium-deficient state, but remained within the laboratory reference range and therefore did not represent hypercalciuria. At follow-up, TRP was calculated as $[1-(\text{urinary phosphate}\times\text{serum creatinine})/(\text{serum phosphate}\times\text{urinary creatinine})]\times100$ using paired values of 40 mg/dL, 0.48 mg/dL, 2.5 mg/dL, and 65.8 mg/dL, respectively, yielding 88%. TmP/GFR was not reported because a baseline paired sample was unavailable and the collection conditions required for reliable interpretation of the follow-up value were not documented (Table 3).

**Table 3:** Laboratory findings after one year of treatment. ALP: alkaline phosphatase; PTH: parathyroid hormone; 25(OH)D: 25-hydroxyvitamin D; TRP: tubular reabsorption of phosphate

Test	Result	Normal reference range
ALP	139 U/L	35-105 U/L
Calcium	8.7 mg/dL	8.6-10.4 mg/dL
Phosphate	2.5 mg/dL	2.5-4.5 mg/dL
PTH	532 pg/mL	15-65 pg/mL
25(OH)D	28 ng/mL	20-50 ng/mL
Spot urinary phosphate	40 mg/dL	No established reference range
Urinary calcium	42.6 mg/24 hours	40-240 mg/24 hours
Spot urinary creatinine	65.8 mg/dL	30-300 mg/dL
Creatinine	0.48 mg/dL	0.55-1.02 mg/dL
Magnesium	2.0 mg/dL	1.6-2.6 mg/dL
TRP	88%	85-95%

Dual-energy X-ray absorptiometry showed Z-scores of -0.6 at the lumbar spine, -3.0 at the femoral neck, and -1.9 at the total proximal femur, indicating bone mineral density below the expected range for age. Vertebral fracture assessment (VFA) showed no vertebral fractures.

At the most recent follow-up, the patient remained clinically stable, with no further progression of her longstanding skeletal deformities during the observed period. Established skeletal sequelae persisted after many years of incompletely characterized disease and treatment before etiologic diagnosis. Renal function remained preserved, and urinary calcium excretion was within the reference range. Renal and urinary tract ultrasonography performed during follow-up was normal, with no evidence of nephrolithiasis or nephrocalcinosis. No treatment-related complications were observed. A formal patient-perspective statement was not collected. Written informed consent was obtained from the patient for the publication of this case and the accompanying images.

## Discussion

25(OH)D is the main circulating form of vitamin D and the most reliable marker of vitamin D status. Its active form, 1,25(OH)₂D (calcitriol), is produced in the kidneys by 1α-hydroxylase (CYP27B1) and regulates calcium and phosphate homeostasis through actions on the intestine, bone, and kidneys [2,3,7,8].

Rickets can be classified into acquired and hereditary forms. Nutritional rickets, the most common type, results from vitamin D deficiency [1]. In contrast, VDDR1A is a rare autosomal recessive disorder caused by biallelic CYP27B1 mutations, impairing 1,25(OH)₂D synthesis despite normal or non-deficient 25(OH)D levels. This results in hypocalcemia, hypophosphatemia, secondary hyperparathyroidism, and defective bone mineralization [2-4,7-14].

Despite well-established clinical and biochemical features, VDDR1A may be difficult to distinguish from more common forms of rickets, leading to delayed diagnosis, as in our patient. Previous studies indicate that earlier recognition and appropriate treatment may reduce the risk of permanent skeletal complications [4,9].

Among hereditary forms, X-linked hypophosphatemia (XLH) deserves particular attention as the most common inherited rickets and shares manifestations with VDDR1A, including growth impairment, lower-limb deformities, delayed motor development, and radiographic rickets [15,16]. Differentiation is essential because their pathophysiology, treatment, and long-term management differ substantially.

Distinguishing VDDR1A from XLH

The main distinction lies in their biochemical profiles and renal phosphate handling. XLH is characterized by renal phosphate wasting, typically with normal serum calcium and normal or mildly elevated PTH. In contrast, VDDR1A is characterized by hypocalcemia, marked secondary hyperparathyroidism, and normal or non-deficient 25(OH)D levels despite active rickets [15,17,18]. In our patient, baseline TRP, TmP/GFR, and serum FGF23 were unavailable, which prevented the direct assessment of renal phosphate handling at presentation. The low absolute 24-hour urinary phosphate excretion was not considered a definitive discriminator because paired serum and urinary phosphate and creatinine values were unavailable. Moreover, the TRP of 88% measured after one year of treatment may have been influenced by therapy and cannot retrospectively establish baseline phosphate handling. The persistent hypocalcemia, profound secondary hyperparathyroidism, and normal 25(OH)D levels nevertheless raised suspicion of VDDR1A rather than XLH. The reported inadequate response to long-term vitamin D₃ supplementation was considered supportive rather than definitive evidence because prior dosing and adherence records were unavailable. Identification of a homozygous CYP27B1 variant classified as likely pathogenic by the testing laboratory, with no additional pathogenic variants identified in FGF23, phosphate-regulating endopeptidase homolog X-linked (PHEX), or the other genes included in the panel, further supported the diagnosis in the clinical and biochemical context.

Clinical clues for early suspicion of VDDR1A

VDDR1A typically becomes apparent between six months and two years of age. Typical findings include growth failure, progressive lower-limb bowing, widening of the wrists and ankles, hypotonia, delayed motor milestones, and bone pain. Although these overlap with nutritional rickets, VDDR1A often presents despite normal or non-deficient 25(OH)D levels [3-5,9-11,19]. Our patient developed early progressive skeletal deformities requiring multiple corrective orthopedic procedures. These findings are consistent with longstanding inadequately controlled rickets, although the relative contributions of initial disease severity, treatment adequacy, adherence, and delayed etiologic diagnosis cannot be reconstructed from the available records.

Red flags for non-nutritional rickets

Early severe disease, persistent skeletal deformities, normal or elevated 25(OH)D levels despite active rickets, marked secondary hyperparathyroidism, and a documented inadequate response to conventional vitamin D supplementation should prompt suspicion of a genetic form of rickets and diagnostic reassessment [1,3-5,9-11]. Our patient reportedly received vitamin D₃ supplementation for more than two decades. Although the absence of dosing and adherence records limits the interpretation of apparent treatment failure, the persistence of hypocalcemic rickets with normal 25(OH)D levels supported etiologic reassessment.

Key biochemical differentiation

VDDR1A and nutritional rickets share hypocalcemia, hypophosphatemia, elevated ALP, and secondary hyperparathyroidism. However, nutritional rickets is characterized by low 25(OH)D, whereas VDDR1A typically presents with normal or elevated 25(OH)D and reduced or inappropriately low 1,25(OH)₂D due to impaired 1α-hydroxylase activity. This dissociation is an established diagnostic clue in VDDR1A [1-3,5,11,19]. In our patient, 1,25(OH)₂D was not measured. Therefore, this dissociation could not be directly demonstrated. Nevertheless, the combination of active hypocalcemic rickets, marked secondary hyperparathyroidism, and normal 25(OH)D levels supported further investigation for impaired vitamin D activation.

Genetic testing and counseling

Molecular testing should be considered early in patients with rickets presenting in infancy, atypical biochemical findings, normal or non-deficient 25(OH)D levels despite active disease, or a documented inadequate response to adequate conventional vitamin D therapy. Early genetic evaluation can confirm the etiology, guide appropriate treatment, and facilitate family counseling, although treatment should not be delayed while awaiting genetic results. When testing is unavailable, clinical and biochemical findings and response to calcitriol may guide management [3-5,9-11,19]. Given the autosomal recessive inheritance of VDDR1A, genetic counseling is recommended for family screening, recurrence risk assessment, and, when appropriate, prenatal diagnosis [20-22]. In our patient, genetic testing was limited to the proband, and parental testing and segregation analysis were not performed. Therefore, parental carrier status and biparental inheritance of the CYP27B1 variant could not be confirmed, and the underlying basis for homozygosity in the absence of reported consanguinity could not be determined.

Other genetic forms in the differential

VDDR1B, caused by CYP2R1 variants, impairs hepatic 25-hydroxylation and is generally associated with low 25(OH)D. VDDR2A results from VDR variants and causes resistance to calcitriol, while VDDR2B involves abnormal VDR-DNA signaling. VDDR3 results from gain-of-function CYP3A4 variants that accelerate vitamin D metabolite degradation [2,3,18]. The panel directly evaluated CYP2R1 and VDR and also included genes associated with other calcipenic and phosphate-related disorders, as listed in the "Case presentation" section. No additional pathogenic variants were detected. However, HNRNPC and CYP3A4 were not included, so the panel did not molecularly exclude VDDR2B or VDDR3.

A practical diagnostic approach

Initial evaluation of suspected calcipenic rickets should include serum calcium, phosphate, ALP, PTH, and 25(OH)D. Low 25(OH)D favors nutritional rickets, whereas normal or elevated levels or failure to respond to adequate vitamin D replacement should raise suspicion of hereditary forms. Measurement of 1,25(OH)₂D may support the diagnosis when available, although access to this assay may be limited. A limitation of our case was the inability to measure 1,25(OH)₂D because of limited availability and high cost. In such settings, biochemical and clinical response to calcitriol may provide supportive therapeutic evidence [3-5,9-11,19], but it does not substitute for the direct measurement of 1,25(OH)₂D or a variant-specific functional assay.

From a molecular perspective, CYP27B1 variants include missense, nonsense, frameshift, and splice-site variants with variable effects on 1α-hydroxylase activity and clinical severity. Complete loss of enzyme function is typically associated with earlier and more severe disease, whereas some missense variants retain partial activity and produce milder phenotypes [4,5,10,23-25]. The homozygous CYP27B1 p.Thr325Met variant identified in our patient was classified as likely pathogenic by the testing laboratory. However, the same variant was previously reported in a patient with monogenic rickets and classified as a variant of uncertain significance based on PM2, PP3, and PP4 criteria [26]. The laboratory report considered additional evidence, including its extreme rarity, evolutionary conservation, deleterious in silico predictions, previous identification in homozygosity, internal observations in three additional affected individuals, and phenotype concordance. Nevertheless, the individual ACMG/AMP criteria supporting an upgrade to likely pathogenic were not provided, and no segregation or variant-specific functional analysis was available. Therefore, the laboratory classification cannot be independently confirmed from the available evidence. In this patient, the homozygous variant was considered supportive of the diagnosis when interpreted together with the early-onset clinical phenotype, biochemical findings, and response to calcitriol, which were compatible with previously described VDDR1A presentations [4,5,9-11,19]. However, the variant should not be regarded as independently demonstrating pathogenicity.

From a therapeutic perspective, nutritional rickets responds to vitamin D and calcium supplementation, whereas VDDR1A requires active vitamin D analogs, such as calcitriol, with calcium supplementation. Previous studies have shown that early sustained treatment promotes biochemical normalization and may limit additional skeletal deformity [5,9-11,19]. In our patient, one year of calcitriol and mineral supplementation was associated with normalization of serum phosphate, improvement in serum calcium, and reductions in PTH and ALP. Urinary calcium increased during treatment but remained within the reference range, consistent with increased calcium availability following the correction of the calcium-deficient state. Persistent PTH elevation indicated incomplete biochemical control. During the observed follow-up, the patient remained clinically stable, with no vertebral fractures, nephrolithiasis, nephrocalcinosis, or other identified treatment-related complications.

The main differences between the forms of rickets are summarized in Table 4.

**Table 4:** Comparison of the key clinical, biochemical, and therapeutic features of nutritional rickets, VDDR1A, and XLH. Symbols: ↓, decreased; ↓↓, markedly decreased; ↑, increased; ↑↑, markedly increased. The findings shown represent typical patterns and may vary according to disease severity, stage, and treatment. VDDR1A: vitamin D-dependent rickets type 1A; XLH: X-linked hypophosphatemia; 25(OH)D: 25-hydroxyvitamin D; PTH: parathyroid hormone; ALP: alkaline phosphatase; 1,25(OH)₂D: 1,25-dihydroxyvitamin D The table was created by the authors based on the following references: [1-4,15-18].

Feature	Nutritional rickets	VDDR1A	XLH
25(OH)D	↓	Normal	Normal
Calcium	Normal/↓	↓	Normal
Phosphate	↓	↓	↓↓
PTH	↑	↑↑	Normal/↑
ALP	↑	↑	↑
1,25(OH)₂D	↓/normal	↓↓	↓/normal
Age at presentation	Infancy/childhood	Usually 6-24 months	Usually childhood
Response to conventional vitamin D alone	Improves	Inadequate	No response
Treatment	Vitamin D + calcium	Calcitriol + calcium	Conventional therapy (phosphate + calcitriol) or burosumab

From a clinical outcome perspective, previous studies have shown that VDDR1A generally has a favorable prognosis when diagnosed and treated early and that delayed treatment may be associated with permanent short stature and skeletal deformities [4,9,27]. In our patient, longstanding deformities persisted, while approximately one year of targeted treatment was associated with sustained biochemical improvement and clinical stabilization. Because historical disease severity, treatment adequacy, and adherence could not be fully reconstructed, this single case cannot determine the extent to which delayed etiologic diagnosis contributed to the irreversible skeletal findings.

## Conclusions

VDDR1A should be considered in patients with early-onset rickets who exhibit normal or non-deficient 25(OH)D levels, hypocalcemia, marked secondary hyperparathyroidism, and an inadequate response to conventional vitamin D therapy. Persistent disease despite appropriate supplementation should prompt diagnostic reassessment and consideration of hereditary causes, with molecular findings interpreted alongside the clinical and biochemical evidence. Differentiating VDDR1A from other forms of rickets is essential because their mechanisms and treatment differ. In this patient, initiation of calcitriol and mineral supplementation was followed by improved mineral metabolism and clinical stabilization over approximately one year, while longstanding skeletal deformities persisted. The case supports earlier etiologic evaluation but does not independently establish that calcitriol prevented progression or that diagnostic delay alone caused the irreversible skeletal sequelae.
